# “Children are a blessing from God” – a qualitative study exploring the socio-cultural factors influencing contraceptive use in two Muslim communities in Kenya

**DOI:** 10.1186/s12978-020-0898-z

**Published:** 2020-04-03

**Authors:** Batula Abdi, Jerry Okal, Gamal Serour, Marleen Temmerman

**Affiliations:** 1 United Nations Population Fund Tanzania country Office, Zanzibar, Tanzania; 2 Ghent University, Ghent, Belgium; 3 Population Council, Nairobi, Kenya; 4 International Islamic Center for Population Studies and Research, Al Azhar University, Cairo, Egypt; 5 International Centre for Reproductive Health, Department of Public Health, Ghent University, Ghent, Belgium; 6 Centre of Excellence Women and Child Health, Aga Khan University, Nairobi, Kenya

**Keywords:** Family planning, Islam and contraception, Culture and religion

## Abstract

**Background:**

Family planning (FP) is one of the high impact public health interventions with huge potential to enhance the health and wellbeing of women and children. Yet, despite the steady progress made towards expanding access to family planning, major disparities across different regions exist in Kenya. This study explored the socio cultural factors influencing FP use among two Muslim communities in Kenya.

**Methods:**

A qualitative study involving Focus Group Discussions (FGDs) and In-depth Interviews (IDIs) was conducted (from July to October 2018) in two predominant Muslim communities of Lamu and Wajir counties. Open ended questions explore key thematic areas around knowledge, attitudes and understanding of contraception, perceived FP barriers, and decision making for contraceptives, views on Islam and contraception, and fertility preference. All interviews were conducted in the local language, recorded, transcribed verbatim and translated into English. Data was analyzed using thematic content analyses.

**Results:**

Although Islam is the predominant religion the two communities, perceptions and belief around FP use were varied. There were differing interpretations of Islamic teaching and counter arguments on whether or not Islam allows FP use. This, in addition to desire for a large family, polygamy, high child mortality and a cultural preference for boys had a negative impact on FP use. Similarly, inability of women to make decisions on their reproductive health was a factor influencing uptake of FP.

**Conclusion:**

Misinterpretation of Islamic teaching on contraception likely influences uptake of family planning. Cultural beliefs and lack of women’s decision power on fertility preferences were a key inhibitor to FP use. Countering the negative notions of FP use requires active engagement of religious leaders and Muslim scholars who are in position of power and influence at community level.

## Plain English summary

The ability of families and individuals to decide freely, for themselves, whether, when, and how many children they want to have is a basic human right. However many women in developing countries are not able to exercise this basic right for various reasons. This study sought to understand social cultural factors that influence the utilization of FP in two Muslim communities in Kenya. The findings show that misinterpretation of the Islamic teaching on family planning is one of the reasons why women are not using family planning. Further the study shows that social cultural values and norms on; desired family size; men marrying many wives; families losing many children due to childhood illness and preference for boy child were seen as hindrance to family planning use. Similarly women’s inability to make their own decision on matters of FP was another deterrent. Engaging religious leaders and Muslim scholars to educate the community and dispel myths on family planning and Islam is very important.

## Introduction

Family planning is one of the high impact public health interventions. Studies have shown that access to family planning services in countries with high fertility rates has the potential to significantly reduce poverty and hunger, as well as avert maternal and childhood deaths [1, 2]. However, many women who would like to delay their next birth or stop childbearing altogether cannot access this important services. Worldwide, 214 million women of reproductive age have unmet needs for family planning (defined as wanting to stop or delay childbearing but are not using any method of contraception) [3–5]. Further, women with an unmet need for modern contraception account for 84% of all unintended pregnancies in developing regions [5].

Kenya has made steady progress in improving access to family planning services. In the past decade, the proportion of married women using a contraceptive method –defined as Contraceptive Prevalence rate (CPR) – increased from 32% in 2003 to 39% in 2009 and to 58% in 2014 [6]. Similarly, unmet need for contraception has slowly but steadily declined, from 28% in 1998 to 26% in 2009 and 18% in 2014.

[6]. Despite the impressive growth in CPR over time, huge disparities exist between counties, with CPR ranging from 2% in Wajir to 76% in Kirinyaga County. Similar disparity exists in fertility levels, ranging from 8 to 2 children per woman in Wajir and Kirinyaga Counties, respectively [6, 7].

Existing evidence shows that several social and cultural barriers impede access to contraceptive services. These include women’s fear of contraceptive side effects, disapproval by partners, lack of knowledge about the contraceptive methods, religion, minimal or lack of spousal communication, and misconceptions [3, 8, 9]. Furthermore, contraceptive uptake is influenced by women’s autonomy and levels of education [10]. Similarly, maternal education has proven to enhance uptake of contraceptives. Research shows that education has a knock-on effect on age at first marriage and entry to the paid labor market, which correlates with reducing fertility [11–13].

Critically, religion has historically raised debate on whether contraceptives should be used. However, from the Islamic perspective, evidence from different authoritative sources suggest that Islam does not forbid the use of contraceptives. For example, the first source of Islamic sharia^1^ (law), the Quran, specifically recommends that mothers breastfeed for two complete years *“and mothers should suckle their children for two whole years...” (Qur’an 2:233)* [14]*.*Scholars describe the 2 years of breast feeding mentioned in the Quran as a means of child spacing to give the mother adequate time to recover from childbirth and care for the child [15–17]. The 2 years of breastfeeding mentioned in the Quran also concurs with the World Health Organization (WHO) recommendation on birth spacing [18]*.* Similarly, the Sunnah, a documentation of the prophet Peace Be Upon Him (PBUH) tradition indicates that coitus interruptus or withdrawal (al Azl) method was practiced during the time of the prophet (PBUH). Many Muslim scholars have used analogical reasoning (qiyas), the third source of Islamic sharia, to legitimize reversible contraceptive methods because both coitus interruptus and modern methods prevent conception. All the four Schools of Islamic Jurisprudence^2^ agree that permanent methods are not permissible without medical justification [16, 19, 20]. Despite enormous evidence and Fatwa^3^ showing the permissibility of reversible contraceptive methods in Islam within the confines of marriage, some Muslim leaders oppose FP in totality. Their objection to FP is based on the following premise: the recommendation in Islam to have many children; beliefs that children are adornment of life and a gift from God; producing children is the purpose of marriage and family planning contradicts the will of Allah and his ability to provide [16, 19]. Those who oppose use of contraceptives cite the following verses from the Quran (Quran 17:31;18: 46) [14]. “And kill not your children for fear of want. We shall provide sustenance for them as well as for you. Verily the killing of them is a great sin.” (Qur’an 17:31) “Wealth and children are the adornment of the life of this world.” (Qur’an 18:46) However, neither of these two verses talks about contraception, but rather about value of children and the obligation to protect their lives.

Although some believe that Islam opposes contraception, no valid explanations exists as to why contexts sharing similar religious values have different CPRs. For example, Wajir and Lamu counties are predominantly Muslim regions; however, these two sites have a stark CPR of 2 and 42% respectively. Apart from Kenya Demographic and Health Survey (KDHS) there has been limited evidence on factors affecting uptake of FP among the muslim comunities in kenya. It is against this background that our study examines how socio-cultural factors influence uptake of family planning.

## Methods

### Study design

This was a qualitative study involving Focus Group Discussions (FGDs) and In-depth Interviews (IDIs) among the Muslim communities in Lamu and Wajir counties. The design was considered suitable for gaining in-depth explanations of the prevailing perceptions and practices regarding contraceptive use and the socio- cultural factors that influence contraceptive use.

### Study setting

Wajir County is part of the former North Eastern province. The county boarders Mandera County to the North, Garissa to the South, Isiolo and Marsabit Counties to the West. The neighboring countries include Somalia to the East and Ethiopia to the North West [21]. The county has a population of 661,941 of which, 298,175 are females and 363,766 are males [22]. It has the lowest CPR in the country at 2% and highest Total Fertility Rate (TFR) of 8 children per woman [6]. The county is ranked as one of the poorest in the country with 76% of the population having no formal education and only 4% having completed secondary level of education or higher [23].

Lamu County is part of the former coast province, located on the Northern coast of Kenya. The county borders Garissa County to the North, Tana River County to the South West, Somalia to North East, and Indian Ocean to the South [24]. It has a population 101, 539 people, 48,494 females and 53,045 males [22]. The county has a CPR of 42% and TFR of 4 children per woman. Nearly a third of the county residents live below poverty line, with only 13% of the resident having completed secondary education [23].

Wajir and Lamu Counties were selected to provide diversity of context for contraceptive uptake, as they both have high Muslim populations, but different levels of contraceptive prevalence.

### Study participants and sampling

Prior to the study, community engagement was done in both counties by working with the local chiefs and community elders. The participants were purposefully selected from three sub-counties, namely Wajir east and Wajir North in Wajir County, and Lamu west in Lamu County. The FGD participants were selected based on sex, age and residence while IDI participants were selected based on their knowledge on socio-cultural practices, religious teaching and their role within the community. Muslim men aged 18–54 and Muslim women aged 15–49 living in study area and willing to participate were targeted for interview. The participants for FGDs and IDI were identified with the help of community leaders, health officers and members of the sub-county health management teams.

A total of 11 FGDs (Wajir *n* = 6 and Lamu *n* = 5) were conducted. FGDs were composed taking age, sex, and position in the community (e.g., religious leader) into account, such that in each county there was an FGD for young women (under age 24 years), older women (above age 30 years), younger and older men (under 24 years, over 30 years), and religious leaders.

A total of 93 participants were recruited to participate in 11 FGDs (7 with men and 4 with women). A description of FGD and IDI participants characteristics is included in Table 1. There were 13 IDIs (Wajir, *n* = 7 and Lamu, *n* = 5) IDI respondents included Islamic scholars, community leaders including women leaders and health workers. In general more men than women were recruited because some of the roles this community are enacted by men (for example most religious leaders and scholars were men with no woman occupying/holding such position. This study was conducted as part of a larger quantitative survey with women in Wajir and Lamu Counties.

**Table 1:** Characteristics of study population

Characteristics	Focus Group Discussion			In-depth interviews		
	Number	Wajir	Lamu	Number	Wajir	Lamu
Male	54	32	22	7	4	3
Female	39	18	21	6	3	3
**Age**						
16–19 years	7	3	4			
20–24 years	9	4	5			
25–29	16	8	8			
29–35	24	14	10	3	2	1
36–40	18	9	9	4	2	2
41–49	19	11	8	6	3	3
**Education**						
No education	36	21	15			
Primary	30	18	12			
Secondary above	27	12	15	13	7	6
**Occupation/Role**						
Unemployed	22	12	10			
Casual job	20	10	10			
Farmer/pastoralist	8	8	–			
Business	11	6	5			
Fishermen	8	–	8			
Civil servant				2	1	1
Health worker				5	2	3
Religious leader	24					
Islamic scholar				2	1	1
Community leader				4	2	2
Total FGD participants 93				Total IDI participants 13		

### Data collection

Data was collected between July and October 2018. Open-ended semi-structured question guides were used to explore participants’ knowledge of contraception, perceived barriers, attitudes, decision making regarding contraceptive use, views about Islam and contraception and fertility. Discussions and interviews were conducted in Somali and Swahili languages for Wajir and Lamu Counties, respectively, and later translated to English. The average duration of the FGDs and IDI was 45 min to 1 h. Interviews were conducted by a team of experienced qualitative researchers supervised by the first author. Data was collected until saturation was reached and no new information emerged during daily study team debriefing meetings. FGDs were conducted in a central place agreed upon with the participants. The FGDs for men and women were conducted separately given the sensitivity of the research topic. The IDIs were conducted in private places of the respondents’ choice. The lead author of the study, fluent in both languages, supervised the data collection process.

### Ethical consideration

Ethical approval for the study was obtained from the Research Ethics Committee of the Aga Khan University, Nairobi (2016/REC-56 (v3)). We also obtained a research permit from the National Commission for Science, Technology and Innovation (NACOTI/P/18/14340/20946) to conduct research activities in the community. All participants provided verbal consent after being informed about the objective of the study. Considering the cultural sensitivities, literacy levels and precedent set by other researchers it was deemed appropriate to obtain verbal consent followed by signature from the research team verifying that consent was indeed taken.

Regarding minors below the age of 18 years, only those who were considered emancipated minors (in this case married adolescents were considered as mature/emancipated minors who could provide their own consent) participated in the interviews. Minors were included in the interviews because early marriage is widely practiced in the two counties. It was therefore necessary to understand the perceptions of married adolescents regarding the challenges in accessing family planning services.

### Data analysis

Data from the FGDs and IDIs were recorded and transcribed verbatim and translated into English. The transcripts were validated for accuracy, through a set review process involving validation by two separate transcribers. We analyzed the data using thematic content analyses, in which a set of codes were developed based on the interview tools, and the emerging themes from the discussions. A code sheet was developed and used for coding the transcripts in ATLAS.ti (Version 7). Data coding was done by two coders, who consulted with the lead author closely, to ensure consistency the codes created were discussed and agreed upon by the team. After the coding process, further analysis was done by grouping texts in analytic categories such as demographics, site, interview type (IDI or FGD) and also by grouping thematically related codes into a code family, in order to gain a broader and deeper understanding of the issues discussed within that theme. The themes were compared across the transcripts and specifically the different analytic categories, to establish the range and similarities of the participants’ perceptions, experiences and views. Review and validation was done by comparing the emerging themes, discrepancies were discussed and consensus was reached. Verbatim quotes used to illustrate the text and effectively communicate its meaning.

## Results

We present findings from FGD involving 93 discussants, and 13 IDI of which 65% were men and 35% were women. Over a third (38%) of the respondents had no education, 31% had completed primary and only 31% completed secondary education and above.

Table 1 shows details characteristics of the respondents.

The findings from this study identified three main themes as the key socio- cultural factors influencing FP uptake: divergent interpretations of Islamic teaching on FP, fertility preferences, gender dynamics and decision-making around contraception.

### Islam and family planning: the divergent opinions

Narratives from Lamu and Wajir both showed divergent views among Islamic scholars, religious leaders, and women and men from the community. Most of the religious leaders and scholars in both the locations asserted that FP (especially child spacing) is supported by Islam. They argued that nowhere in the Quran and the Sunnah prohibited FP. Furthermore, they referred to specific evidence in the Sunnah which showed that contraception was allowed, particularly, the use of the withdrawal method (*Al azl*). The following quotes illustrate views that Islam supports FP/ child spacing:

The Sunnah does not directly talk about child spacing as such but it talks about the prophet (PBUH) noticing that his companions doing practicing coitus interruptus (Al azl) and when he heard about it he did not forbid this practice therefore his followers concluded that if it was anything that is not allowed in Islam he could have stopped it immediately. The intention of Al azl was to prevent pregnancy just like the modern temporary methods". - (**IDI, Islamic Scholar,-Wajir)**

Our religion says that God is the one who gives us children, but on another perspective, we are not allowed to burden ourselves. To do family planning is okay, what is wrong is abortion. Its better you have a child that you can take care of". **(IDI woman leader- Lamu)**

However, most respondents from Wajir believed that FP was strictly limited to child spacing for 2 years only. They implied that women should give birth after every 2 years between pregnancies until menopause. Often the responses referred to the Quranic recommendation that mothers breastfeed their children for 2 years to restore their physical and psychological wellbeing before another pregnancy. Respondents from Wajir also alluded that women will not get pregnant during the 2 years of breastfeeding since they have lactational amenorrhea (absence of menstruation during breastfeeding).

In our religion in the Quran we were told for the woman who gives birth she should breast feed her child for 2 years so that both the mother and the baby’s health will not be affected but if what you are talking about is child spacing more than those two years unless it is for medical reason then the religion does not allow, even ALLAH says in his book give birth and do not think about poverty because he is the provider". **(FGD Religious leader- Wajir)**

“Most people believe that when women are breastfeeding they will not get pregnant” **(women leader, wajir)**

Additionally, some respondents believed that FP was only acceptable under certain circumstances. For example, they alluded that FP was permissible if the health of the woman has deteriorated or the woman has had several caesarian sections such that another pregnancy would be detrimental to her health.

Islam allows family planning only if it’s child spacing and not stopping the women from giving birth completely and also the other reason is that if a doctor recommends family planning for a woman for a reason related to her health and the baby, the religion has no objection. (**FGD, Religious leader- Wajir**,)

On the contrary some respondents opined that Islam does not support FP, pointing out the contradiction between FP and the principles of God as the sustainer and provider. Such respondents frequently quoted the Quran to back their claims that children are a blessing from God and that each child comes with their own provisions (rizq).

“Family planning is haram because you are preventing a living creature from coming to the world.” **(Male FGD-Lamu)**

“Because they have the belief that God is the one who provides and sustains so family planning is a sin to them even when we are giving them health talk some say we the health workers are irrational, they ask why do you want us to kill the unborn child, they tell us taking the pill is to kill the child.” (**IDI health worker- Wajir)**

Interestingly, although there are those who believed that Islam is in contradiction of the use of FP, they justified using contraceptives because of the economic situation which they said prevents them from sustaining the desired large family, a common situation in Lamu.

“Islam doesn’t allow family planning, it is haram, (forbidden) we are just practicing because of the difficult economic times, even the Prophet said fill the world with children so that I have a big ummah (society) in the day of judgement”.- (**FGD women, Lamu)**

### Fertility preferences and contraceptive uptake

Understanding fertility preference of a community is fundamental for family planning programmes. With regards to fertility preference many sub-themes emerged: desired family size and contraceptive use, influence of child mortality on contraception, role of polygamy, son preference and contraceptive use. The desire for more children among men in Wajir stems from the cultural value of children in a predominantly nomadic society where children are a form of wealth and provide labor force. The desire for more children as expressed by men in Wajir has a direct impact on contraceptive use.

#### Desired family size and contraceptive use

Women in both Lamu and Wajir counties desired a “modest” family size of between 4 and 6 children. Men in Lamu also desired a similar family size of between 4 to 5 children; however, most of their counterparts in Wajir desired as many as 15 children.

“In our community we want our wives to give birth until the menopause stage that is what I want personally. If you ask a number, I can tell you personally I need many children, around 15, because nobody wants few number when it comes to children”- (**Male FGD- Wajir)*.***

“In our community we want our wives to give birth until the menopause stage that is what I want personally … My reason is am following the practice of the prophet and the prophet said I want my followers to reproduce and fill the earth”- (**Male FGD_ Wajir)**

On the other hand, respondents in Lamu justified their desire for a smaller family size because of the economic burden of sustaining larger families.

***“****I would say four is enough. Many women in this current time, they are employed women and the economy doesn’t favor one having many children. Some say they want four, others even seven children, but most prefer four because it is manageable.” –(***IDI woman leader- Lamu)**

#### Child mortality and contraceptive use

Both counties had similar views on child mortality and contraception. Having many children was a mitigating factor in the high prevalence of child mortality. Respondents view a large family size as a coping mechanism for child mortality. The results show that child mortality affects uptake of contraceptive use in two ways; when a child dies, the mother will stop using contraceptives to have another child and families tend to have more children considering that some could die in their childhood, thus affecting contraceptive use.

“We prefer many children so that when some die at least you will still have some. For example, when you have only one or two and God takes them what will you do? So it’s wise you bear as many children as you can.” (**FGD women –Lamu)**

“many people have that perception that I rather have many children so that when some die, still you have other children.” (**IDI Women leader- Wajir)**

#### The role of polygamy in contraceptive use

Across the sites most respondents supported the view that polygamy (a practice in which a man has more than one wife), is accepted in Islam. With greater importance attached to procreation, polygamy seems to accelerate child births and fuel competition among co-wives to have more children. Competition among co-wives likely leads to more pregnancies among women in polygamous relationship thus leading to low contraceptive use. This phenomenon was more frequently reported by respondents in Wajir as opposed to Lamu.

“*On my personal judgement, polygamy is one of the drivers of not using contraceptives as the women may compete having many children.” (****IDI health worker- Wajir).***

“In polygamous marriage when a woman is married to a rich man many children will help her to inherit more wealth.”( **IDI woman leader- Wajir )**

#### Preference for sons and contraceptive use

Generally, most respondents mentioned a preference for male children. This finding was more pronounced in Wajir than Lamu County. Consequently, respondents in Wajir, affirmed that having a boy is an honor. Thus, many women with only daughters will avoid FP at all costs in the hopes of having a boy.

“…the other one is cultural bias towards the male… you will see a mother has 4 girls she sees this is a good number but her wanting to get a boy child, she will keep trying to get pregnant until she is able to get a boy or until menopause.”- (IDI **Health worker- Wajir)**

“The society values boys more than girls, so if you don’t have a boy you keep giving birth until you are able to get [one].” (**women FGD-Wajir)*.***

### Gender relations and decision-making regarding contraceptive use

In patriarchal societies, men are the sole decision makers, and they also dominate sexual and reproductive issues including FP use. In the two communities, the perception of the man as the head of the family led most women to believe that men had authority on the decisions about contraceptives use. Nevertheless, a few of the respondents disagreed, stating that this decision should be jointly made by couples after a healthy discussion. The ability of women to make their own decisions on how many children to have and how often directly correlated with high/low contraceptive use. The quotes below illustrate how respondents viewed FP decision-making. Furthermore, the discrepancy between desired family size between men and women in Wajir shows that often men achieve their fertility desires regardless of their partner’s choices.

“In many cases, the decision of how many children the family needs lies with the husband. In some instance[s], the decision is made by the couple. In few instances, advice from the doctor due to the health of the mother.” (**FGD men- Lamu)**

“Mostly, you will find it is the man who makes the decision and sometimes they don’t care. I think there are very few families who sit down and say, “Ok, how many children do we want to have?” But if there is one of them who were to make decision it is usually the man.” (**Islamic Scholar- Wajir)**

In some instances mothers-in-law have a big influence a couple’s decision to use contraception. These narratives show the influence mothers-in-law have on contraceptive use.

*“Mother-in-law also suggests, and most of the time they can’t be ignored because they have so much influence in their son’s lives.’” (****FGD women- Lamu)***

“When other people like mothers-in-law are involved, this issue about family planning she will not accept; she will even curse you….**” (FGD women- Wajir)**

#### Spousal communication and contraceptive use

Couple communication is a very important factor in contraceptive decision-making and utilization. However, respondents had varied opinions on couple communication on contraceptive use. In Lamu respondents reported that to some degree, there are conversations on contraceptive use, and while in Wajir similar discussions were rare or non-existent but often associated with the education level of the couples. As depicted in the quotes below spousal communication appears to happen more frequently in Lamu than Wajir.

“We talk to our husbands, some support the idea while others don’t support the idea.” (**FGD women- Lamu)**

*“At the family level discussing family planning is an issue [for only the few] who are educated because there is mass which are over 80 percent of uneducated people the issue of a family planning it is not a topic of discussion…”* ( **IDI health manger- wajir )**

However, some respondents reported covert use of contraceptives, likely due to of lack of communication. Those who reported covert use of contraceptive mostly attributed partner and other family member opposition to contraceptive use.

“There are some who say they hide the pills because if their husbands find out they end up getting divorced but there are those who are clever enough they come for injection that way the husband will not know.” (**IDI women leader-Wajir)**

“In cases where men don’t support, this forces the woman to practice family planning in secrecy. This can also lead to relationship wrangles and [divorce].” (**FGD- Lamu women)**

## Discussion

This study explored the sociocultural factors that influence use of family planning among Muslim communities in Lamu and Wajir counties, Kenya. The counties vary substantially in terms of rates of poverty, level of education, and utilization of modern contraceptives. Interestingly, the findings highlight that the residents of the two counties also hold divergent interpretations of Islamic teaching on family planning, role of fertility preferences in contraceptive uptake and gender dynamics and decision-making on FP uptake.

The position of Islam on contraception has been a key subject of debate, centered on the permissibility of family planning in Islam, with important consequences for contraceptive uptake in Muslim communities. As one of the few empirical studies in sub-Saharan Africa exploring sociocultural factors influencing uptake of family planning among Muslim populations, this study provides important insight into how varies interpretations of Islam intertwine with other cultural values to produce support for, or opposition to, modern contraceptive use. Specifically, findings highlight how cultural values have been labeled as religious teachings, with implications for contraceptive uptake.

Notably, our study shows that many Muslim scholars and leaders in both counties agree that FP is accepted in Islam. They accept it within the context of marriage and, more specifically, to aid with the spacing and timing of pregnancies. However scholars interviewed noted that permanent and non-reversible contraceptive methods without medical justification are not permissible. These findings correlate with the broader consensus on the acceptability of FP within Islam and are consistent with the documentations in the Islamic doctrine [17, 19, 25–27].

In the debate around Islam and contraception, the advocates of FP have used many citations from the Quran and Sunnah, the first and second sources of Islamic sharia, respectively. The Quran mentions that Allah desires ease for us *(Quran 2:185) and “Allah tasks not a soul beyond its capacity (or limits).” (Quran 2:286)* [14]*.* Furthermore, proponents of FP have used evidence from Hadith and Sunnah show that FP is permitted in Islam, and that Al Azl (withdrawal method) was used during the time of the prophet (PBUH); based on analogical deduction (i.e the third source of Islamic Sharia (Qiyas)) modern FP methods are allowed [19]. The citations represent Islam as a religion of mercy and moderation [16, 19].

However, our findings show an apparent disconnect between the understanding of FP from the above citation of Islamic teaching and knowledge and practices of FP in the community. This disconnect is influenced by conflicting opinions regarding FP, with most men and women interviewed considering the use of FP as ‘haram’ (forbidden). This belief might be a key driver of the low uptake of FP, especially in Wajir. Moreover, many respondents believe that FP is only allowed in special circumstances such as, when a woman is ill or has had a caesarian section, meaning that by the community standards many women will not “qualify” to use FP. Another misinterpretation which could influence the attitude and use of FP is that Islam recommends only 2 years of breast feeding for child spacing. Many of the respondents interviewed highlighted this philosophy by stating that mothers have lactational amenorrhea (absence of menstruation during breastfeeding) and therefore will not conceive during this period. This perception was more common in Wajir as opposed to Lamu, which may explain the difference in uptake of FP and high TFR. In a similar study, Mir and Shaikh argue that misconception in Islamic teachings contributes to the low utilization of contraception [25, 28–30]. To see change, Muslim scholars and religious leaders must demystify religious and cultural myths and misconceptions. Furthermore, given their influence, different studies [25, 28, 31] have highlighted the importance of working with Muslim scholars and religious leaders to yield positive results in FP uptake.

Those who viewed FP as unacceptable in Islamic culture justified their beliefs through several factors: the view that contraceptives kill the unborn child, that every child comes with their own blessing and provision (rizq) and that the prophet (PBUH) urged his followers to produce and fill the earth. Non- use of FP is therefore based on the view that it will infringe on the principles of Islam of directing one’s trust towards Allah (tawakkul), the provider and sustainer. However, Omran argues that the idea that FP contravenes the principles of tawakkul requires further analysis. Omran posits that FP does not breach the ‘tawakkul’ concept, but instead suggests that we need to see FP as doing what is humanely possible within the context of what is willed by Allah [19]. Another possible reason for this misunderstanding of Quran and Hadith is the language barrier. The Holy Quran and Hadith are written in Arabic. Even with accurate translation, people may misunderstand the verses and need clarification by religious authorities fluent in Arabic. This has been the experience of the Al Azhar mission to several counties in Kenya, including Lamu in 2015 [32].

The second theme to emerge in this study is fertility preference. Having insights into people’s fertility intentions helps to understand child bearing norms, as well as improving the design for behavioral change interventions of FP programmes. Research from Kodzi and others has shown a significant relationship between past intentions of having additional children and future fertility [33]. The findings show that the desire for more children was informed by many factors, including: sociocultural values attached to large family size, child mortality, and polygamy and son preference.

In relation to ideal family size, our study shows a striking difference between the two counties with regards to desire for larger families, influenced by cultural and religious belief. The demand for larger family size, as expressed by men in Wajir, could be one of the key drivers of non-use of contraception in Wajir County compared to Lamu. The preference for large families has mainly been expressed by men in the study, particularly men in Wajir County. They base their preference on the belief that more children constitute more wealth, prestige and from a religious perspective that every child is a gift from God, with their own provisions and blessings. These findings are consistent with what has been documented previously [33–35]. Furthermore, a study in Kenya, looking at male fertility preference found that men in North Eastern region (wajir is in this region) desire more children three times higher as compared to men in Nairobi [36]. This is also evident from the finding of KDHS, which shows the TFR for Wajir is double (7.8) that of the national average 3.9 [6].

The women in both counties are predominantly controlled by a strong patriarchal system, which encourages polygamous marriages. There is cultural importance given to female fertility and women in the communities are expected to bear as many children as possible. Consequently, women in polygamous marriages compete with co-wives regarding the number of children they conceive to acquire more of the husband’s wealth. Further, the findings show remarkable difference between the counties with regards to polygamy which could be another factor influencing the low uptake of contraceptive in Wajir compared to Lamu. While the men rationalized polygamy both from a religious and cultural perspective, it is also an honor, prestige, and a sign of wealth to have many wives and children. Other studies have shown that men use polygamy to attain their fertility goals independent of their individual wives [37].

The findings of this study show a relationship between child mortality and contraceptive uptake. Given the high child mortality in the two counties, bearing more children is used as a coping mechanism to compensate for the death of a child. In these situations, women will discontinue a contraceptive method when a child dies or have more children as a forward-looking strategy. This link between child mortality and fertility concurs with other research [38, 39]. In addition, our findings indicate that son preference drives high fertility, especially in Wajir, where women will continue giving birth until they get a son thus, and affecting contraceptive use. The preference for sons is informed by the patriarchal norms that sons are agents for family continuity and lineage as evidenced by studies elsewhere [36, 40].

The degree to which women exercise their decision-making powers about their health and lives is shaped by the social intuitions around them. The patriarchal system of most African households means that women continue to be relegated to low status within their communities [41, 42]. The findings confirm the inability of women to make decisions about family planning; some resort to using FP in secret for fear of their husbands or mother-in-laws. Our finding on women’s inability to decide the number, timing and spacing of their children which directly impact contraceptive use, agrees with others studies [10, 41, 42].

Lack of communication on matters of contraception was also a key issue highlighted in the study. Research has shown that couple communication is positively linked to uptake of family planning [43]. Furthermore, our findings show limited couple communication on matters of contraception. Lack of spousal communication was more prevalent in Wajir County, which could further explain the low uptake of FP in the county.

Although the study has revealed some interesting findings on the social cultural factors influencing uptake of FP, it is worth noting that the study had its own limitations. The findings documented may not be generalized among the Muslim communities given the diverse social cultural backgrounds. While there are fundamental differences with other faith denominations, we believe our approach and lessons learned can contribute broadly and inform expanded research on faith based perspectives and family planning. Due to the limited scope this study we did not explore how observed differences in CPR across the two counties is attributed to differential interpretations of Islam in the context of differences in levels of poverty, education, pastoralism, child mortality between the two counties – all of which are known to affect contraceptive utilization. Furthermore this study was not powered to compare whether respondents’ answers varied by age, education and parity which have been shown to be correlated with family planning uptake and fertility desires.

This an understudied area and to our knowledge this study is among the first few to explore the role of religion particularly Islam on family planning uptake in sub-Saharan Africa. Therefore, there is need to explore the role of Islamic teaching as an important factor to consider along with the other sociocultural elements when designing and implementing family planning programmes in similar settings. In the last decade the FP programmes have focused on system strengthening and addressing structural barriers around the supply and demand. However to achieve the global and national FP targets it is imperative that we understand and address the complexity around FP and sociocultural barriers particularly religion and design culturally appropriate FP programmes. Given the role of men at family level as key decision makers it is critical to understand better men’s fertility preference. Therefore, there is need for further research on men fertility desires and its implication for contraceptive use especially in Wajir County.

## Conclusion

This study analyzed information from community members in the two counties with diverse background as described in the result section. Our findings shows that three key factors influencing the uptake of FP. The misinterpretation of Islamic teaching on contraception has negatively influenced uptake of family planning. Similarly, fertility preference, influenced by social cultural values that encourage many children, has also hindered use of FP. Further, gender dynamic and decision making on contraception play a pivotal role in determining use of contraceptives, with women’s inability to make decisions about family planning being the major deterring factor.

In order to address the low uptake of FP especially in Wajir, it is critical to engage religious leaders and Muslim scholars to demystify myths and misconceptions around FP and Islam. It is worthwhile to learn from progressive Muslim countries that have made strides in family planning and dialogue at the county level with key stakeholders on family planning. Given the role of education in decision-making on contraceptive use, it is imperative for the national and county government to invest in education, particularly for women and girls to enhance their ability to make informed decisions. Finally the findings of this study can be used to develop culturally appropriate social behavior change materials on family planning with active engagement of religious leaders.

## Data Availability

The datasets used and/or analyzed in the study are available from the corresponding author on reasonable request.

## Abbreviations

CPR: Contraceptive prevalence rate
DHS: Demographic and health survey
FP: family planning
FGD: Focus Group Discussions
IDI: In-depth Interviews
KDHS: Kenya demographic and health survey
TRF: Total fertility Rate
PBUH: Peace Be Upon Him.

## Footnotes

1 **Sources of Islamic law (Shariah)** -The primary sources of Shariah are the Quran (Islam’s holy book) and the Sunnah, the sayings and deeds of the Prophet Mohammad and his Companions. Also based on the Quran and Sunnah but subordinate to them are two other sources for Shariah: the consensus of Islamic jurists (ijmae) and analogy (qiyas).

2 **Schools of Islamic Jurisprudence**-The schools of Islamic jurisprudence are called madhahib, which means “paths” or “ways.”. All schools of jurisprudence consider the Quran and the Prophet’s tradition (Sunnah) as their primary sources. They differ only in relation to some interpretations, the validity of other sources of jurisprudence, and the methods of formulating a ruling. The four schools of thought are; Hanafi, Maliki, Shafei and Hanbali

3 Fatwa- authoritative legal opinion or learned interpretation by a qualified jurist regarding issues pertaining to Islamic Shariah.
